# Compromised COPII vesicle trafficking leads to glycogenic hepatopathy

**DOI:** 10.1242/dmm.050748

**Published:** 2024-09-30

**Authors:** Yuxi Yang, Xue Zhang, Qingshun Zhao, Jingzi Zhang, Xin Lou

**Affiliations:** ^1^ Medical School, Nanjing University, Nanjing 210093, China; ^2^ Research Center for Life Sciences Computing, Zhejiang Laboratory, Hangzhou 311100, China

**Keywords:** COPII vesicle, Liver disease, Glycogenic hepatopathy, Thyroid hormone, Zebrafish

## Abstract

Being a vital cellular process, coat protein complex II (COPII) vesicle trafficking has been found to play a crucial role in liver metabolism. However, its functions and the underlying mechanisms in systemic metabolic homeostasis have not been fully understood. Here, with a newly identified gene trap zebrafish line (*sec31a^nju221^*), we show that compromised COPII vesicle trafficking leads to biphasic abnormal hepatic metabolism. During the larval stage, deficiency of COPII-mediated trafficking leads to activation of the unfolded protein response and the development of hepatic steatosis. By using epistasis analysis, we found that the eIF2α–ATF4 pathway serves as the primary effector for liver steatosis. In adult *sec31a^nju221^* fish, the hepatosteatosis was reversed and the phenotype switched to glycogenic hepatopathy. Proteomic profiling and biochemical assays indicate that *sec31a^nju221^* fish are in a state of hypothyroidism. Moreover, our study shows that thyroid hormone treatment alleviates the metabolic defects. This study provides insights into processes of liver diseases associated with vesicle trafficking impairments and expands our understanding of the pathological interplay between thyroid and liver.

## Teaser

**Summary:** The *sec31a*-mutant zebrafish model provides insights into the processes of glycogenic hepatopathy associated with vesicle trafficking impairments and reveals the pathological interplay between the thyroid and liver.

## INTRODUCTION

Serving as an ancient and essential route in eukaryotes, the coat protein complex II (COPII) mediates forward trafficking of protein and lipid cargoes from the endoplasmic reticulum (ER) to the Golgi apparatus (Bethune and Wieland, 2018; Zanetti et al., 2011). The liver houses many metabolic processes, including the production and secretion of lipids and proteins based on physiological conditions; thus, COPII vesicle trafficking in the liver is of particular importance in systemic metabolic homeostasis. Clinical studies have shown that variants in COPII components or accessory factors are associated with a wide spectrum of metabolic disorders, ranging from isolated recurrent acute liver failure to a multisystemic phenotype (Garcia-Cazorla et al., 2022; Yarwood et al., 2020). Analyses of animals carrying COPII mutations have started to provide us with critical information about why a similar trafficking defect can cause different diseases (Lu and Kim, 2020). However, owing to the complex interactions among tissues and organs, as well as the presence of COPII paralogs, many pieces of this puzzle are still missing.

As a key component of the outer coat, Sec31a (and its paralog Sec31b) binds to Sec13 to form heterotetramers, and these complexes constitute the edges of the of COPII cage (Gurkan et al., 2006). Genetic studies on human patients demonstrated that variants in *SEC31A* could cause pronounced neurological abnormalities along with defects in other organs (Halperin et al., 2019). Given that reported cases are scant and no vertebrate genetic model has been reported so far, the pathological features led by Sec31a deficiency have not been fully characterized and analyzed.

Characterized by excessive accumulation of glycogen in hepatocytes, glycogenic hepatopathy is an underdiagnosed liver complication seen in patients with diabetes, eating disorders, Dumping syndrome and other maladies (Kransdorf et al., 2016; Resnick et al., 2011; Umpaichitra, 2016). The clinical manifestations of glycogenic hepatopathy include hepatomegaly, abdominal pain and elevated liver enzyme levels (Khoury et al., 2018; Sherigar et al., 2018). The pathophysiology of glycogenic hepatopathy is poorly understood, partly hindered by the absence of suitable animal models. Currently, researchers speculate that it is the consequence of recurrent wide fluctuation in both glucose and insulin levels (Sherigar et al., 2018). Additionally, there has been limited exploration into the involvement of other metabolism-regulating organs, such as the thyroid, in its development.

In the current study, we provide *in vivo* evidence demonstrating that compromised COPII vesicle trafficking leads to biphasic abnormal hepatic metabolism. We identified a gene trap zebrafish line, *sec31a^nju221^*, which bears a hypomorphic mutation of *sec31a*. Phenotyping analysis showed that the unfolded protein response (UPR) was specifically activated in the liver of *sec31a^nju221^* larvae and hepatic steatosis was induced. Epistasis analysis based on gene knockouts was performed and the eIF2α–ATF4 pathway was found to serve as the primary effector for liver steatosis. In the livers of adult *sec31a^nju221^* fish, the phenotypes switched to a drastic decrease of lipid droplet deposition and an excessive accumulation of glycogen granules. The integration of proteomic profiling and biochemical assays indicated that *sec31a^nju221^* fish are in a state of hypothyroidism. Furthermore, we performed a rescue experiment and found that supplementation of thyroid hormone could partially reverse the hepatic metabolic defects in *sec31a^nju221^* fish. The data presented in this work demonstrate that *sec31a^nju221^* fish can serve as a vertebrate genetic model for glycogenic hepatopathy. This study also provides mechanistic insights into liver disease processes and previously undescribed thyroid–liver pathological interactions caused by vesicle trafficking deficiency.

## RESULTS

### Compromised COPII-mediated transport causes steatohepatitis in zebrafish embryos

**Fig. 1.**
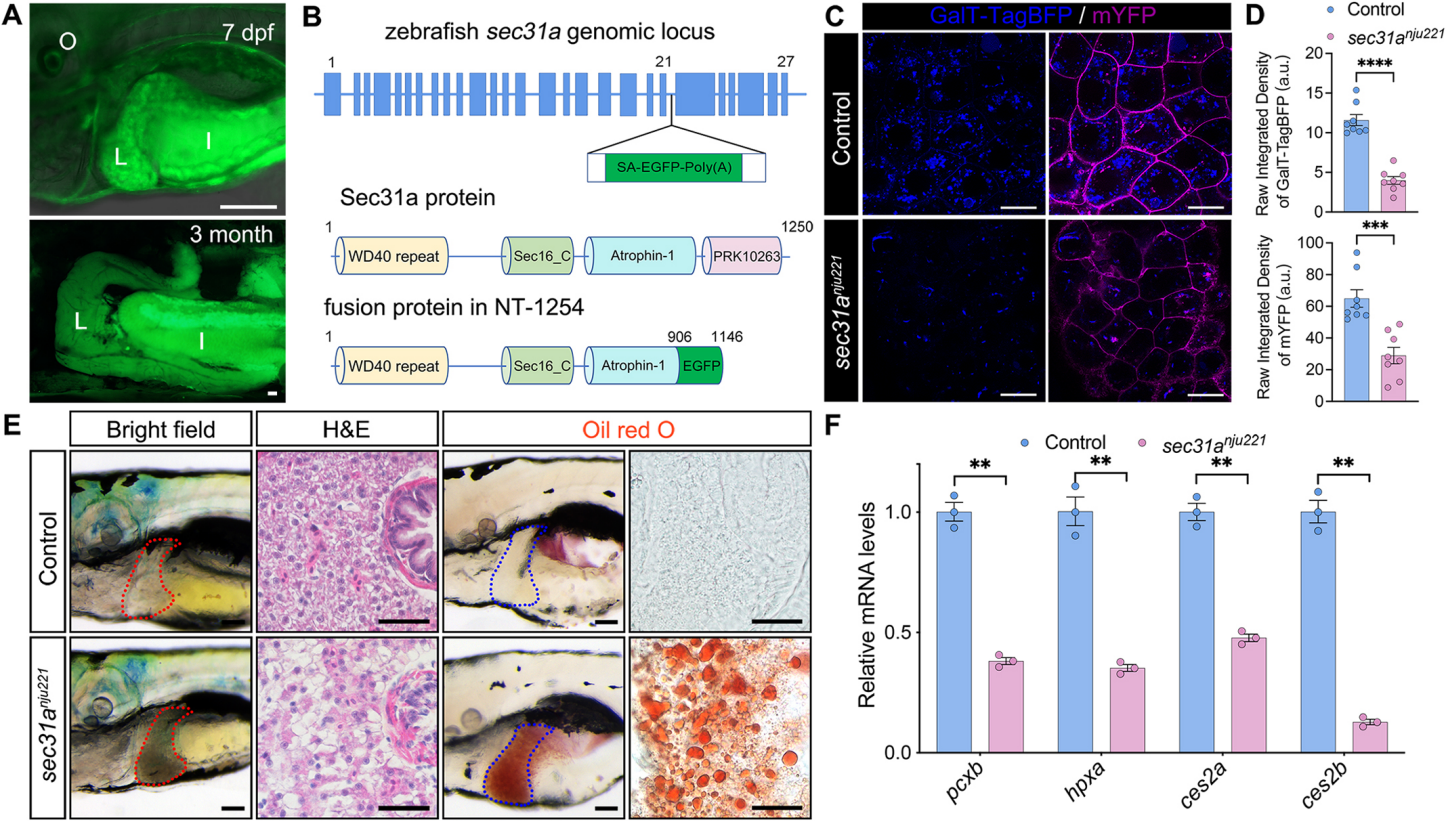
**Deficiency of COPII-mediated trafficking in *sec31a^nju221^* mutants leads to the development of hepatic steatosis.** (A) GFP expression pattern in the zebrafish trapping line NT-1254 at the indicated stages. Lateral views, head to left. I, intestine; L, liver; O, otic capsule. Images are representative of eight fish at each stage. Scale bars: 200 μm. (B) Upper panel: zebrafish *sec31a* genomic locus. The transposon is inserted into the 21st intron. Lower panel: schematic representations of the domain structure of the zebrafish Sec31a protein and the fusion protein in NT-1254 fish. (C) Live-cell imaging of shield stage zebrafish embryos injected with the trans-Golgi network reporter GalT-BFP (blue) and the plasma membrane reporter mYFP (magenta). Scale bars: 20 μm. (D) Quantification of GalT-BFP and mYFP fluorescence intensity (*n*=6). a.u., arbitrary units. ***P<0.001; ****P<0.0001 (unpaired two-tailed Student's *t*-test). (E) Left: bright-field images depicting the liver of live zebrafish larvae. Middle: Hematoxylin and Eosin (H&E) staining of liver from 7 dpf zebrafish larvae. Right: Oil Red O staining of 7 dpf zebrafish larvae. For bright-field and ORO staining images, livers are outlined with dashed lines. Scale bars: 100 μm (bright-field images); 20 μm (H&E and Oil Red O images). Images are representative of 20 fish within each group. (F) Quantitative PCR analysis of RNA samples extracted from livers of 7 dpf larvae. Data are mean±s.e.m. **P<0.01 (unpaired two-tailed Student's *t*-test).

In a Tol2 transposon-mediated gene trapping screen to search for previously unidentified regulators of animal development (Hou et al., 2017), we identified the NT-1254 zebrafish line, in which the GFP reporter demonstrated a dynamic expression pattern with strong signals evident in liver from 72 h post fertilization (hpf) that persisted into adulthood (Fig. S1; Fig. 1A). 5′ RACE (rapid amplification of cDNA ends) was used to identify the gene trapped in the NT-1254 line and the sequencing result indicated that the gene-trapping element was integrated within the 21st intron of the *sec31a* locus, resulting in a transcript that encodes a fusion protein lacking 352 amino acids on the C-terminal of Sec31a (Fig. 1B). At 24 hpf, homozygous mutant embryos from in-crosses of heterozygous fish showed shorter yolk extension and defects in fin fold growth (Fig. S2). To confirm that mutation of *sec31a* represents the causal event in the phenotype, mRNA encoding wild-type Sec31a was injected at the one-cell stage and the morphological defects in homozygous mutant embryos were efficiently rescued (Fig. S2); thus, we designated this trapping allele as *sec31a^nju221^* and use *sec31a^nju221^* to refer to homozygous mutants. As Sec31a encodes an essential component of the COPII coat, we monitored protein trafficking in *sec31a^nju221^* embryos using fluorescent protein reporters: GalT-BFP destined for the trans-Golgi network and mYFP destined for the plasma membrane. Live images acquired with confocal microscopy showed that in *sec31a^nju221^* embryos, the signal intensity of both reporters at target compartments was greatly reduced compared to that in the wild-type siblings (Fig. 1C,D), suggesting that COPII-mediated transport is compromised. Sec31a and Sec31b interact with Sec13 to form heterotetramers that serve as the edges of the COPII lattice, and it has been reported that mutation in the zebrafish *sec13* gene leads to malformation of the skeleton cartilage and hypoplasia of digestive organs (Niu et al., 2012). Interestingly, the development of the head skeleton and digestive organs was largely normal in *sec31a^nju221^* embryos, except for slightly delayed calcification of cranial bones and defective formation of the intestinal epithelium (Fig. S3). Co-immunoprecipitation analysis also revealed that the Sec31aΔC352-GFP fusion protein partially retained the ability to form homodimers and bind with both wild-type Sec31a and Sec13 (Fig. S4). These results indicated that *sec31a^nju221^* is a hypomorphic mutation, enabling us to examine the phenotypic consequences of compromised COPII-mediated trafficking at later stages.

By 5 days post fertilization (dpf), *sec31a^nju221^* larvae exhibited a dark-colored liver (Fig. 1E), which suggested steatohepatitis. Histological analyses of *sec31a^nju221^* larvae showed ballooning of hepatocytes with an amassed presence of macrovesicles, and Oil Red O (ORO) staining revealed extensive accumulation of lipid droplets in the liver (Fig. 1E). We also found that the expression of genes involved in key hepatocyte processes was decreased in *sec31a^nju221^* larvae, such as *pcxb* (involved in carbohydrate metabolism), *hpxa* (involved in iron transport), and *ces2a* and *ces2b* (involved in xenobiotic metabolism) (Fig. 1F). Taken together, these data indicate that impairment of COPII-mediated trafficking in *sec31a^nju221^* larvae leads to the development of hepatic steatosis, which is accompanied by decreased liver function.

### The eIF2α–ATF4 pathway acts as the primary mediator for liver steatosis in *sec31a^nju221^* larvae

In order to determine the effect of deficiency of COPII-mediated trafficking, low-input RNA sequencing (RNA-seq) was performed using livers dissected from 5 dpf larvae. In the livers from *sec31a^nju221^* larvae, the expression of a total of 1486 genes was significantly altered (515 downregulated genes and 971 upregulated genes) (fold change >2, false discovery rate <0.001) (Fig. S5A). Gene Ontology (GO) enrichment analysis revealed that the differentially expressed genes in *sec31a^nju221^* samples were involved in protein processing and transport, the UPR and lipid metabolism (Fig. S5B). We confirmed the reliability of expression data obtained from low-input RNA-seq by quantitative PCR (qPCR) and found that the change in the expression of representative genes from enriched gene sets was comparable (Fig. S5C). In addition, enrichment analysis in transcription factor targets showed that a significant portion of the upregulated genes are transactivated by mediators of the UPR such as Atf3, Xbp1, Atf6 and Srebp1 (also known as Srebf1) (Fig. S5D). Furthermore, reverse-transcription PCR showed robust *xbp1* mRNA splicing in livers from *sec31a^nju221^* larvae, and no obvious signal change was noted in the remaining parts of the body (Fig. S5E). These data demonstrate that the UPR is specifically activated in the liver of *sec31a^nju221^* larvae.

**Fig. 2.**
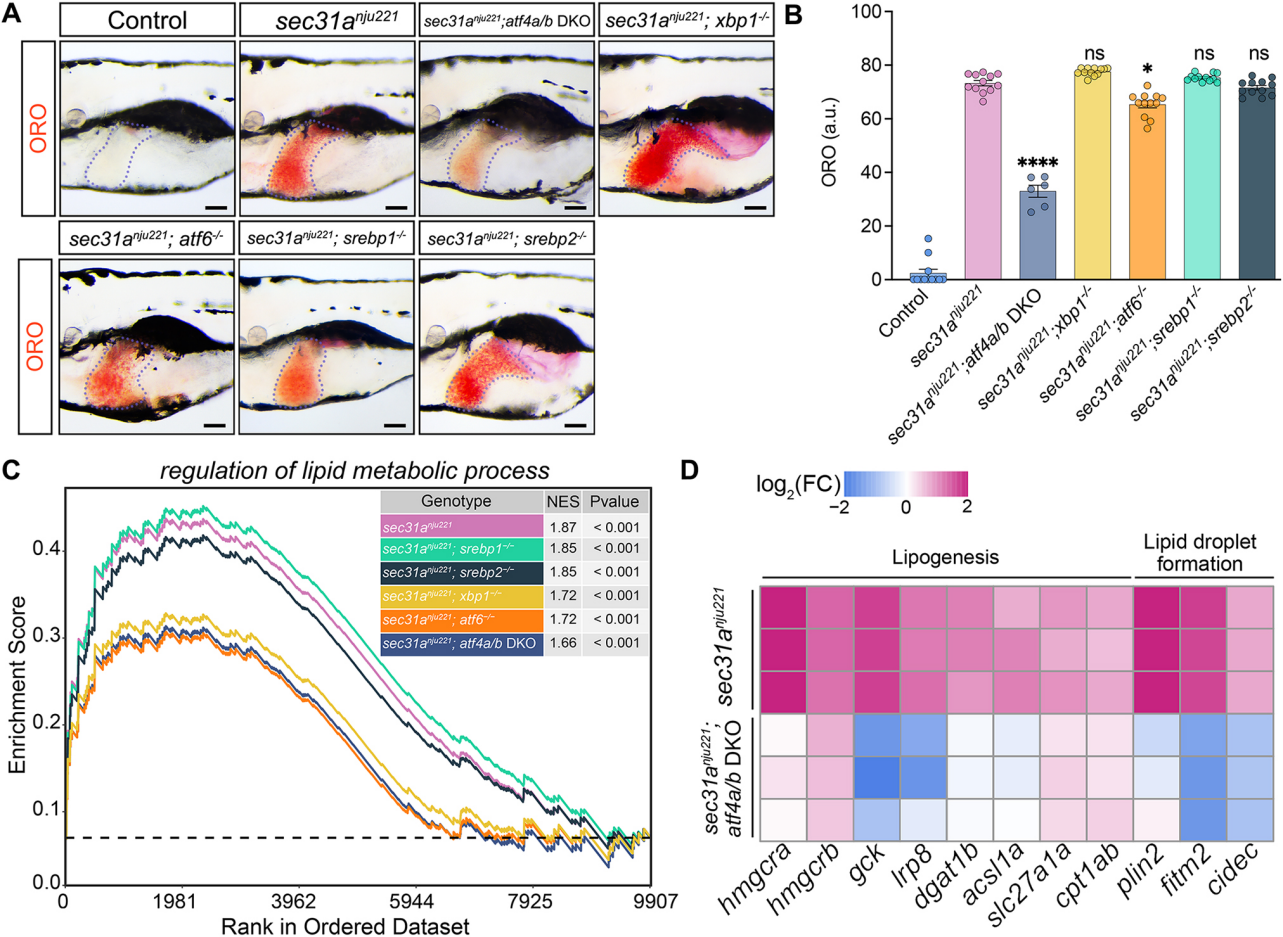
**Epistasis analysis indicates that the ATF4 pathway serves as the main mediator for liver steatosis in the *sec31a^nju221^* background.** (A) Oil Red O (ORO) staining of 7 dpf zebrafish larvae. Livers are outlined with dashed lines. Scale bar: 100 μm. (B) Quantification of ORO staining in livers as shown in A. Each data point represents an individual animal with bars representing mean±s.e.m. Comparison between groups was undertaken using one-way ANOVA with Tukey's multiple comparisons test. For A,B, *n*=6 for the *sec31a^nju221^*; *atf4a/b* DKO group, and *n*=12 for all other groups. a.u., arbitrary units. ns, not significant; *P<0.05; ****P<0.0001. (C) Gene set enrichment analysis (GSEA) of RNA-seq expression values from larvae livers showing distinct separation of different genotypes. NES, normalized enrichment scores. Statistical analysis was performed using an empirical phenotype-based permutation test embedded in GSEA. (D) Heatmap showing the expression levels of genes involved in lipogenesis and lipid droplet formation in livers from *sec31a^nju221^* and *sec31a^nju221^*; *atf4a/b* DKO larvae. FC, fold change.

Previous studies have demonstrated that ER stress can induce hepatic steatosis. Various mechanisms, such as SREBP activation, diminished cholesterol secretion and reduced fatty acid oxidation, have been proposed to underlie this pathological change (DeZwaan-McCabe et al., 2017; Jo et al., 2013; Kammoun et al., 2009; Rutkowski et al., 2008). Nevertheless, the specific roles of distinct arms of the UPR and their downstream effectors in the induction of hepatic steatosis have not been thoroughly investigated. To address the involvement of various UPR branches in hepatic steatosis developed in *sec31a^nju221^* larvae, we used CRISPR/Cas9 technology to knock out genes encoding downstream transcription factors: *xbp1*, *atf4a*, *atf4b* and *atf6* (Figs S6-S8). As Srebp1 and Srebp2 (also known as Srebf2), the master transcription factors regulating lipid synthesis, are activated by ER stress in a manner similar to Atf6 (Ye et al., 2000), we also disrupted *srebp1* and *srebp2* to investigate their contribution (Figs S9 and S10). Interestingly, hepatic lipid accumulation in *sec31a^nju221^* larvae was significantly decreased when *atf4a* and *atf4b* (hereafter *atf4a/b*) were depleted simultaneously (Fig. 2A,B; Table S1). When *xbp1* was disrupted, the triglyceride content in the liver of *sec31a^nju221^* larvae was substantially increased, whereas the loss of *atf6*, *srebp1* or *srebp2* showed no noticeable impact on the phenotype (Fig. 2A,B; Table S1).

To gain molecular-level insights into the epistasis revealed by the ORO staining experiment, we dissected livers from these zebrafish lines at 7 dpf and performed low-input RNA-seq. We then conducted gene set enrichment analysis (GSEA) on genes involved in the regulation of lipid metabolic processes across each genotype. Among all genotypes, the *sec31a^nju221^*; *aft4a/b* double knockout (DKO) line yielded the lowest normalized enrichment score, indicating that its transcriptome deviates the least from the wild-type transcriptome (Fig. 2C). GO enrichment analysis indicated dysregulation in lipogenesis and lipid droplet formation in the *sec31a^nju221^* mutant (Fig. S5B). We then examined the effect of *atf4a/b* knockout on these processes. We found that *atf4a/b* depletion abrogated the upregulation of *hmgcra* and *hmgcrb*, which encode rate-limiting enzymes for cholesterol synthesis (Bloch, 1965), as well as of *dgat1b*, *acsl1a* and *lrp8*, which encode proteins involved in triacylglycerol biosynthesis (Yen et al., 2008) (Fig. 2D). In addition, livers from *sec31a^nju221^*; *atf4a/b* DKO larvae expressed lower levels of *plin2*, *fitm2* and *cidec*, which are crucial players in the formation and maintenance of lipid storage droplets (Olzmann and Carvalho, 2019) (Fig. 2D). Positioned at the center of cellular stress signaling, ATF4 proteins are translationally regulated by four eIF2α kinases in eukaryotic cells, each respectively responding to ER stress, amino acid limitation, hypoxia and oxidative stress (Pakos-Zebrucka et al., 2016). To determine whether ER stress or nutrient deprivation causes ATF4 activation in *sec31a^nju221^* fish, we treated *sec31a^nju221^* embryos separately with PERK and Gcn2 (encoded by *eif2ak4*) inhibitors. ORO staining revealed that attenuating the PERK–phospho-eIF2α–ATF4 axis with GSK2606414 mitigated hepatic steatosis in *sec31a^nju221^* fish, whereas inhibition of Gcn2 with GCN2-IN-1 was ineffectual (Fig. S11). This indicates that ER stress is the primary driver of ATF4 activation in *sec31a^nju221^* fish.

Collectively, these results suggested that the eIF2α–ATF4 pathway exerts the primary role in the development of hepatic steatosis in *sec31a^nju221^* larvae. Because *sec31a^nju221^*; *atf4a/b* DKO larvae exhibited a marginal but substantial increase in hepatic lipid content, it is likely that steatosis would not be solely mediated by eIF2α–ATF4 pathway.

### Adult *sec31a^nju221^* escapers display glycogenic hepatopathy

**Fig. 3.**
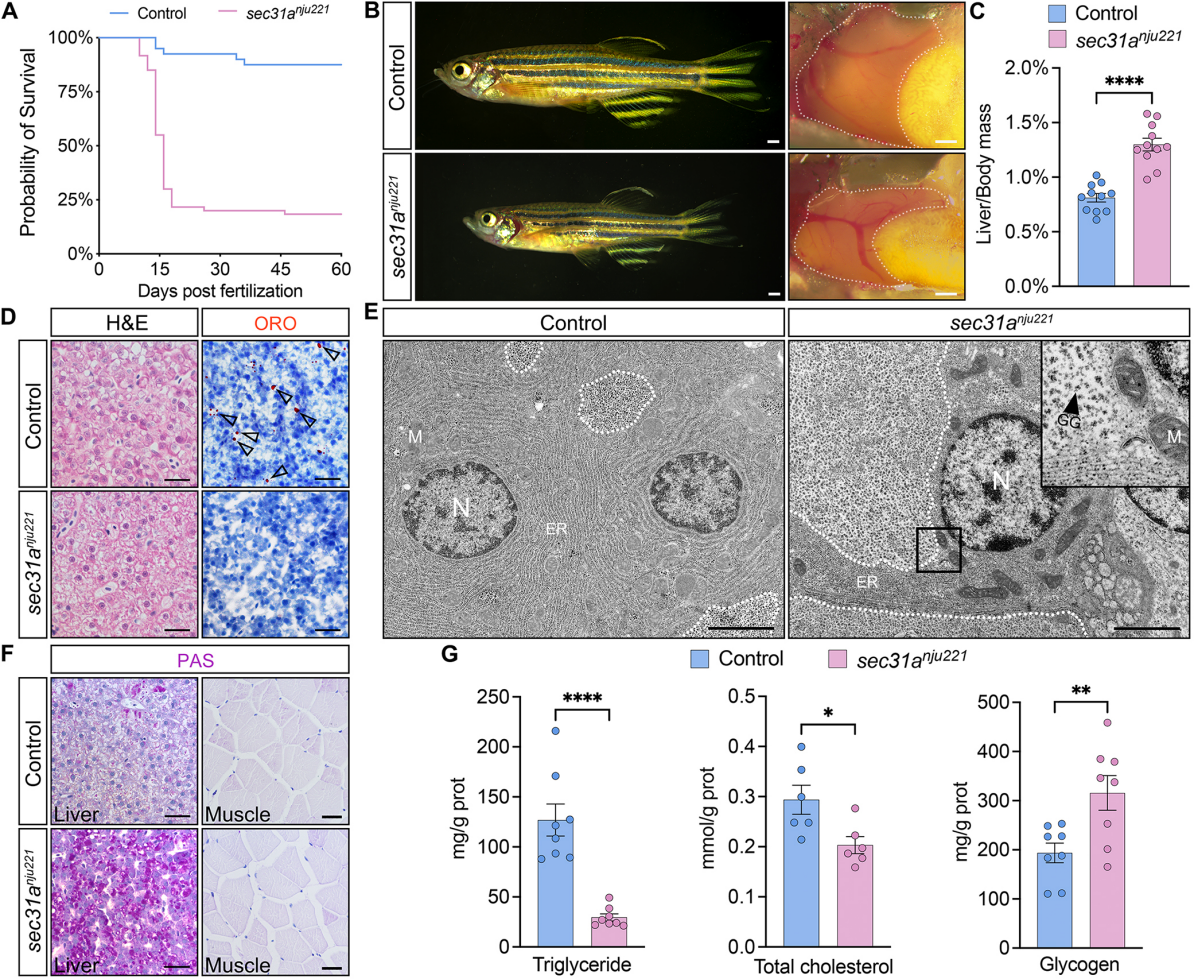
**Adult *sec31a^nju221^* escapers display overt glycogenic hepatopathy.** (A) Representative Kaplan–Meier plot for *sec31a^nju221^* fish and clutchmates from one of three independent experiments. *n*=80 for each group; *P*<0.0001 (Mantel–Cox test). (B) Live images of control and *sec31a^nju221^* zebrafish at 60 dpf. The livers are outlined with dashed lines. Lateral view, anterior to the left. Scale bars: 1 mm (left); 1 mm (right). (C) Ratio of the liver to body mass from 60 dpf *sec31a^nju221^* fish and clutchmates. *n*=11 for each genotype. (D) Representative photographs of H&E and ORO staining. Arrowheads indicate oil droplets. Scale bars: 20 μm. (E) Electron micrographic pictures of liver biopsy from control and *sec31a^nju221^* zebrafish. The distribution of intracellular glycogen granules is outlined with dashed lines. ER, endoplasmic reticulum; GG, glycogen granule; M, mitochondria; N, nucleus. The inset shows an enlarged view of the boxed area. Images are representative of three fish per group. Scale bars: 2 μm. (F) Representative photographs of PAS staining of liver and muscle sections. Images are representative of six fish per group. Scale bar: 50 μm. (G) Triglyceride (*n*=8), cholesterol (*n*=6) and glycogen (*n*=8) levels in fish livers were measured with enzymatic colorimetric assays. Each data point represents an individual animal with bars representing mean±s.e.m. Comparison between groups was performed using unpaired two-tailed Student's *t*-test. *P<0.05; **P<0.01; ****P<0.0001.

Owing to severe hepatic dysfunction, *sec31a^nju221^* fish exhibited substantial mortality during juvenile stages (Fig. 3A). Although afflicted by stunted growth, approximately 22% of *sec31a^nju221^* fish could be raised to adulthood (Fig. 3A,B). These ‘escapers’ offered an opportunity to explore the phenotypic consequences in adults; consequently, we conducted a range of analyses on these animals. Dissection of *sec31a^nju221^* fish revealed that there was an appreciable increase in liver size (measured by liver to body mass ratio, Fig. 3C). Histological analysis of *sec31a^nju221^* liver biopsy samples revealed swollen hepatocytes with cytoplasmic rarefaction and accentuated cell membranes (Fig. 3D). ORO staining showed that *sec31a^nju221^* adult fish had significantly lower hepatic triglyceride and lipid accumulation, which was confirmed by enzymatic colorimetric assays (Fig. 3D,G).

To investigate the mechanisms underlying the histological change in the *sec31a^nju221^* liver, we examined the ultrastructure of hepatocytes with transmission electron microscopy. These images showed a striking increase in intracellular glycogen granules (Fig. 3E) in hepatocytes from *sec31a^nju221^* fish, which was further evidenced by periodic acid-Schiff (PAS) staining and colorimetric assays (Fig. 3F,G). We also examined the levels of glycogen in the skeletal muscle, another primary storage organ for glycogen; no perceivable change was observed in *sec31a^nju221^* samples (Fig. 3F). Collectively, these data indicate that, as development progresses, the hepatosteatosis in *sec31a^nju221^* fish was reversed and the hepatic metabolic phenotype switched to the depletion of lipid droplets and excessive accumulation of glycogen.

### Thyroid hormone treatment alleviates the metabolic defects in *sec31a^nju221^* escapers

**Fig. 4.**
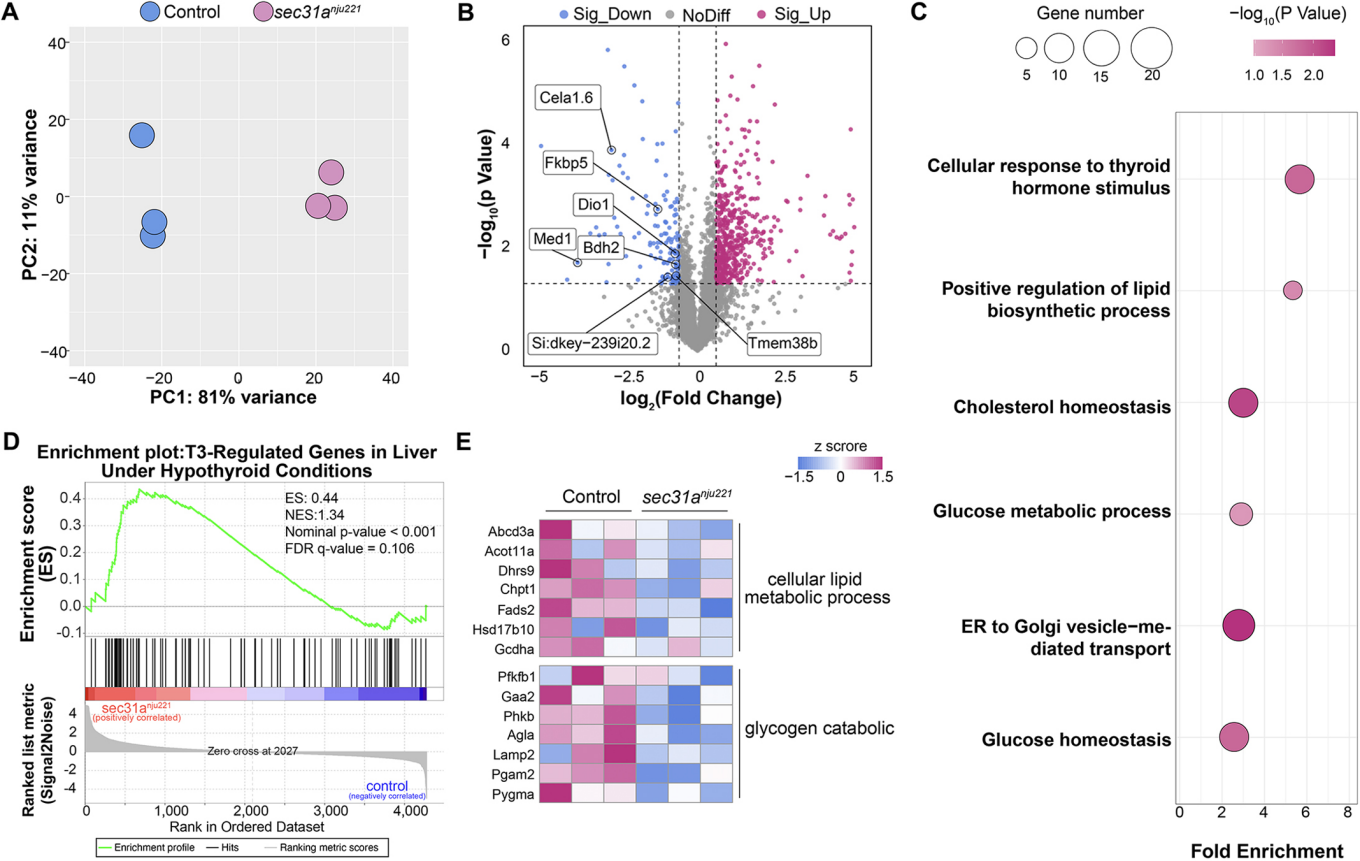
**Proteomics analysis reveal that thyroid hormone signaling is dampened in *sec31a^nju221^* livers.** (A) Principal component analysis (PCA) shows that the mass spectrometric data clearly segregate based on the sample genotype. Three replicates are shown for each genotype. (B) Volcano plot of proteomic data. Fold change in the expression of each protein is shown and the *P*-value was calculated by performing a unpaired two-tailed Welch's *t*-test. The horizontal dashed line indicates *P*=0.05; vertical dashed lines indicate ratios of 0.5 and 2, respectively. Proteins with significant increase in expression levels are shown in magenta, proteins with significant decrease in expression levels are in blue, and proteins with no significant change in expression levels are in gray. Proteins that are decreased downstream targets of thyroid hormone signaling are indicated. (C) Bubble plot showing the enrichment for Gene Ontology terms of differentially expressed proteins in the liver of adult *sec31a^nju221^* fish. The size of each dot represents the number of different genes in the corresponding biological process and molecular function term. (D) GSEA plot of thyroid hormone (T3)-regulated genes in the liver of adult *sec31a^nju221^* fish versus wild-type sibling. FDR, false discovery rate; NES, normalized enrichment score. (E) Heat map of lipid and glycogen metabolism pathway genes dysregulated in the liver of adult *sec31a^nju221^* fish.

In order to elucidate the mechanisms underlying the glycogenic hepatopathy phenotype in adult *sec31a^nju221^* fish, we conducted global protein expression profiling of liver samples using a data-independent acquisition (DIA) approach. As shown in Fig. 4A, an obvious separation trend between the *sec31a^nju221^* and control group could be visualized in the principal component analysis plot. Using fold change >1.2 and *P*<0.05 as filtering criteria, we discovered 805 differential proteins in the *sec31a^nju221^* group (Fig. 4B). GO analysis and GSEA revealed that, along with vesicle-mediated transport, proteins deregulated in *sec31a^nju221^* samples were also involved in thyroid hormone signaling mediation and response (Fig. 4C,D). Close examination of the proteomic data unveiled a considerable downregulation of the reported direct targets (Grontved et al., 2015) of thyroid hormone signaling, specifically those involved in lipid or glycogen metabolism (Fig. 4E). These protein expression data indicated that thyroid hormone signaling was dampened in *sec31a^nju221^* fish.

**Fig. 5.**
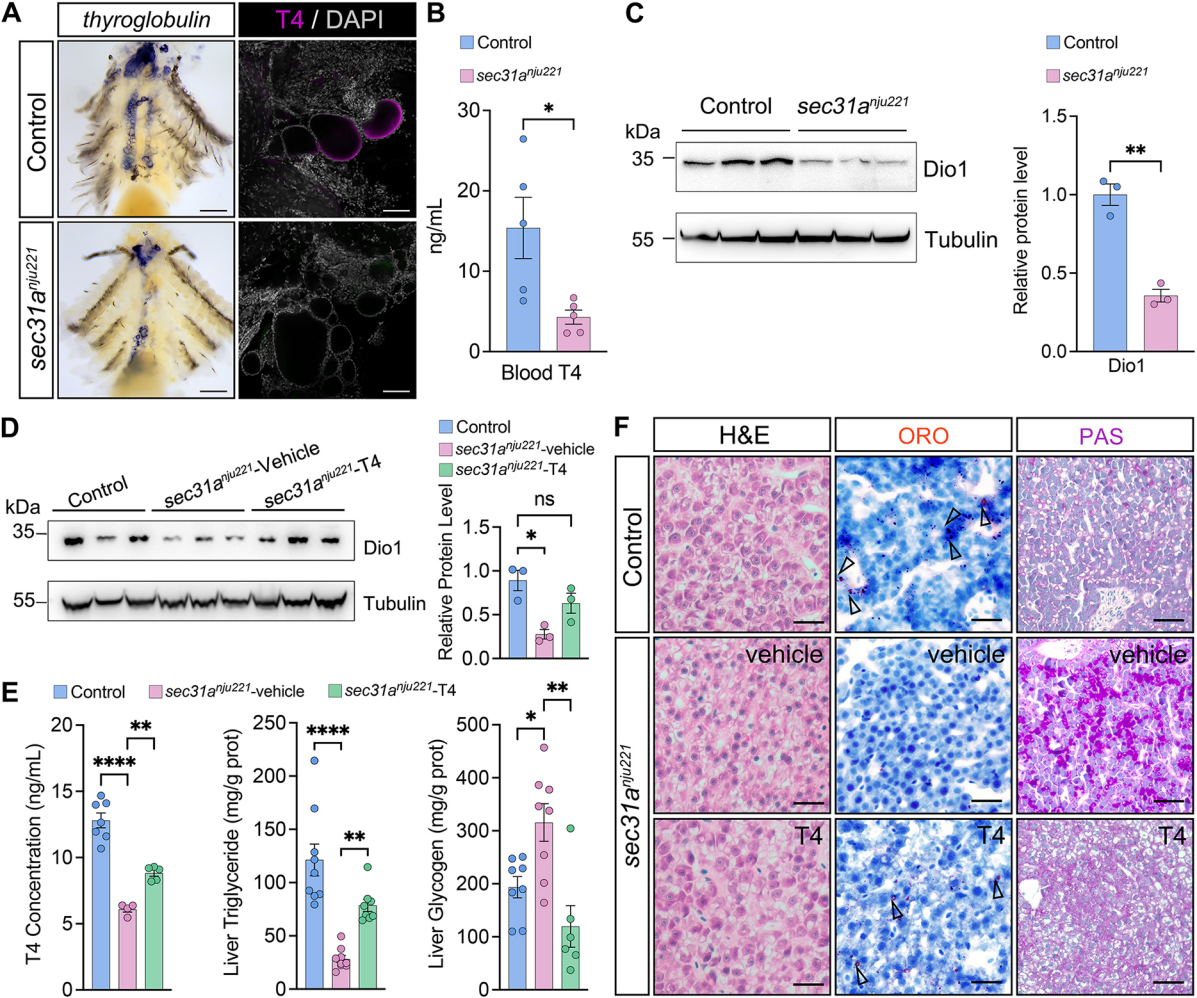
**The hepatic metabolic defects in *sec31a^nju221^* adult fish can be alleviated by thyroid hormone treatment.** (A) Left: whole-mount RNA *in situ* hybridization for *thyroglobulin* in the thyroid gland of adult fish. Right: representative immunofluorescence of thyroxine (T4, purple) in the thyroid follicle lumen of wild-type and *sec31a^nju221^* fish (*n*=5 zebrafish per group). Thyrocyte identity is confirmed by DAPI counterstaining (white). Scale bars: 500 μm (left); 50 μm (right). (B) Serum thyroxine (T4) concentrations in wild-type and *sec31a^nju221^* fish (*n*=5). Data are mean±s.e.m. (C) Western blot analysis and relative quantification of Dio1 from adult wild-type and *sec31a^nju221^* fish liver (*n*=3). (D) Western blot analysis and relative quantification of Dio1 from adult wild-type, vehicle-treated *sec31a^nju22^*^1^ and T4-treated *sec31a^nju221^* fish liver (*n*=3). (E) T4, triglyceride and glycogen levels in fish liver were measured with enzymatic colorimetric assays. *n*=9, 9 and 8 for control, vehicle-treated *sec31a^nju221^* and T4-treated *sec31a^nju221^* fish, respectively. Each data point represents an individual animal, with bars representing mean±s.e.m. Comparisons between groups in B and C were performed using two-tailed unpaired Student's *t*-tests, and comparisons between groups in D and E were performed using one-way ANOVA with Tukey's multiple comparisons test. ns, not significant; *P<0.05; **P<0.01; ****P<0.0001. (F) Representative photographs of H&E, ORO and PAS staining of liver biopsy samples. Arrowheads indicate oil droplets. Images are representative of five fish per group. Scale bars: 50 μm.

As the defective COPII-mediated transport has been found to affect the secretion of thyroglobulin (a prohormone of thyroxine and triiodothyronine) and eventually leads to clinically significant hypothyroidism in humans (Knobel and Medeiros-Neto, 2003), we examined thyroid hormone generation in *sec31a^nju221^* fish. Whole-mount *in situ* hybridization for *thyroglobulin* (*tg*) showed severe hypoplasia of thyroid follicles in *sec31a^nju221^* fish (Fig. 5A). By immunostaining, only a trace mount of thyroxine (T4) was detected in the remaining thyroid follicles of *sec31a^nju221^* fish (Fig. 5A); consistent with this result, colorimetric assay showed that blood T4 levels in *sec31a^nju221^* fish were significantly lower than those in control fish (Fig. 5B). To further confirm the attenuation of thyroid hormone signaling in *sec31a^nju221^* fish, we examined the protein level of type I iodothyronine deiodinase (Dio1), which is an important controller of local thyroid hormone availability and its expression and activity are particularly regulated by thyroid hormone (Gereben et al., 2008). Western blot analysis showed that the Dio1 protein was pronouncedly reduced in the liver of *sec31a^nju221^* fish (Fig. 5C). Collectively, these data indicate that *sec31a^nju221^* fish are in a state of hypothyroidism. Then, we asked whether supplementation of thyroid hormone to *sec31a^nju221^* fish could reverse the observed metabolic phenotypes. Thyroxine treatment was initiated at 3 months of age, when the metabolic phenotypes in *sec31a^nju221^* fish were already apparent. After a sustained 4-week T4 treatment in a closed system, we first confirmed the efficacy of the regimen by examining blood T4 and Dio1 protein levels (Fig. 5D,E), followed by histological and colorimetric assays. We observed increased lipid accumulation (judged by ORO staining) and decreased deposition of intracellular glycogen (judged by PAS staining and enzymatic colorimetric assay) (Fig. 5E,F). Histological analysis of liver biopsy samples also revealed that the rarefaction of the cytoplasm in *sec31a^nju221^* fish was eased in the T4 treatment group (Fig. 5F). Therefore, the hepatic metabolic phenotypes observed in *sec31a^nju221^* fish could be partially rescued through the administration of thyroid hormone.

## DISCUSSION

Disruptions in COPII vesicle trafficking are linked to a broad spectrum of disorders. In the present study, we report that a defect in *sec31a* leads to biphasic abnormal hepatic metabolism. Our data showed that hepatic steatosis was induced in the liver of *sec31a^nju221^* larvae and the phenotypes switched to glycogenic hepatopathy at the adult stage. Further analysis showed that adult *sec31a^nju221^* fish exhibit a state of hypothyroidism, and administration of thyroid hormone alleviated the hepatic metabolic defects, including excessive glycogen accumulation. Owing to the essentiality of the COPII machinery in development, COPII variants in humans are often hypomorphic. Our results suggest that creating animal models with hypomorphic mutations in other COPII genes would help us understand why similar trafficking defects can manifest different clinical features. Additionally, the discovery of this previously unrecognized thyroid–liver pathological interaction enhances our understanding of liver disease processes and may lead to improved strategies for diagnosis and clinical management.

The data presented in the current study revealed an intriguing steatosis to glycogen accumulation phenotype switch in the livers of *sec31a^nju221^* fish. It is probable that during the larval stage, the UPR and its downstream mechanisms are activated to augment lipid content as a short-term resolution for restoring ER homeostasis in hepatocytes, leading to the development of steatosis. Given that unresolved ER stress results in the suppression of the expression of the master regulators of metabolism (Rutkowski et al., 2008), it is plausible that this suppression and the hypometabolic effect of hypothyroidism have double impact, causing a reduction of lipogenesis and the diversion of substrates toward glycogen synthesis in the liver of adult *sec31a^nju221^* fish. Although researchers discovered several decades ago that thyroid hormone deficiency in animals could increase liver glycogen concentration (Snedecor, 1968), the mechanism controlling the shunting of substrates between carbohydrate and lipid metabolic pathways in this setting remains far from clear. As proteomic profiling revealed changes in a number of glycogen metabolism regulators in the liver of *sec31a^nju221^* fish (Fig. 4E), an important question for future work is how these proteins modulate glycogen accumulation in this scenario.

Why the impairment of function in Sec31a only causes prominent defects in the liver is an engaging question. We hypothesize that this results from a combination of tissue-specific demands and compensatory mechanisms. The liver, as the central organ for the production and secretion of lipids and proteins necessary for systemic metabolic homeostasis, relies heavily on vesicle trafficking, making it particularly vulnerable to defects in the COPII system. Additionally, Sec31b, a paralog of Sec31a, has been found to be abundantly expressed during the early stages of zebrafish embryogenesis (Niu et al., 2012). It is possible that less affected tissues have higher levels of Sec31b expression, which could compensate for the loss of Sec31a function, whereas the liver lacks this redundancy. However, future work is needed to confirm the presence of Sec31b protein in the less affected organs.

Notably, hepatic steatosis was induced in *xbp1^−/−^* larvae and the triglyceride content in the liver of *sec31a^nju221^*; *xbp1^−/−^* larvae was significantly elevated. Transcriptome profiling revealed that in contrast to *sec31a^nju221^* samples, lipid oxidation and the export of lipids were inhibited in *sec31a^nju221^*; *xbp1^−/−^* samples (Fig. S12). Xbp1 deficiency could lead to constitutive activation of its upstream kinase/endoribonuclease Ire1α, which then cascades into two signaling outputs: the JNK pathway and regulated Ire1α-dependent decay (Siwecka et al., 2021). It is likely that these mechanisms contribute in parallel to the exacerbated hepatosteatosis in *sec31a^nju221^*; *xbp1^−/−^* fish.

## MATERIALS AND METHODS

### Animals

All animal experimentations were carried out in accordance with approved guidelines of the Institutional Animal Care and Use Committee of Nanjing University. All zebrafish lines were kept on an AB background (see Table S5 for complete list of fish lines used in this study).

### RNA *in situ* hybridization

Transcription of digoxigenin-labeled antisense RNA probes was performed using standard methods. Whole-mount RNA *in situ* hybridization was carried out as previously described (Thisse and Thisse, 2008). *tg* cDNA used for riboprobe synthesis was amplified using the forward primer 5′-AGGTGGAGAATGTTGGTGTG-3′ and the reverse primer 5′-CTCCAACTCTGGCAATGACT-3′.

### Transmission electron microscopy

Adult zebrafish livers were dissected and fixed with 2.5% (v/v) glutaraldehyde in 0.2 M cacodylate buffer (50 mM cacodylate, 50 mM KCl, and 2.5 mM MgCl_2_ pH 7.2) overnight. After washing with cacodylate buffer, tissues were cut into ∼1-2 mm^3^ pieces and immersed in 1% OsO_4_ in 0.2 M cacodylate buffer for 2 h at 4°C. Then, samples were washed and submerged in 0.5% uranyl acetate overnight, dehydrated through a graded series of ethanol, and embedded in resin (Spurr's Low Viscosity Embedding Media Kit; EMS, 14300). Ultra-thin sections were cut using an ultramicrotome and mounted on copper grids. Sections were stained with uranyl acetate and lead citrate, and observed using a transmission electron microscope (HITACHI, H7650).

### Monitoring protein trafficking in zebrafish embryos

To investigate the trans-Golgi network and plasma membrane, plasmids pCS2-mYFP-CAAX and pCS2-GalT-TagBFP were generated. Plasmids were digested and linearized using restriction enzyme NotI (Takara, 1166), mRNA was obtained by using the mMESSAGE mMACHINE SP6 transcription kit (Thermo Fisher, AM1340) and the Poly(A) Tailing kit (Thermo Fisher, AM1350). *In vitro* synthesized capped mRNAs encoding GalT-TagBFP (50 pg) and mYFP (50 pg) were injected at cell stage 1 into wild-type siblings and sec31anju221 zebrafish embryos using the SYS-PV820 Microinjection System (WPI). Injected embryos were cultured under standard conditions at 28°C in fresh E3 medium. At mid-gastrulation (80% epiboly, 8 hpf), live images were acquired using confocal microscopy.

### Generation of zebrafish knockout lines

Guide RNAs were designed using the CRISPR/Cas9 target online predictor (https://cctop.cos.uni-heidelberg.de). 50 pg of single guide RNAs and 500 pg of Cas9 protein were co-injected into one-cell-stage embryos. Sexually mature F0 CRISPR-injected fish were crossed with wild-type fish, and their F1 offspring were screened for CRISPR-directed deletions using PCR and sorted accordingly. CRISPR-positive F1 larvae were then raised to adulthood and crossed again with wild-type fish to establish genetically stable F2 mutant lines. Fin clips were taken from each F2 mutant line, and positive samples were sequenced. Genotyping oligonucleotides used in this study are listed in Table S2.

### Cell culture

The HEK293T cell line was obtained from the American Type Culture Collection, with cells cultured under 5% CO_2_ at 37°C in Dulbecco's modified Eagle medium (Gibco, 11965092) supplemented with 10% fetal bovine serum (Gibco, 10270-106), and passaged with 0.25% trypsin (Gibco, 25200072). The cell line used was mycoplasma free.

### Co-immunoprecipitation

HEK-293T cells were used for co-immunoprecipitation (Co-IP). Plasmids were transfected and a total of 4×106 cells were harvested in 60-mm dishes. Cells were homogenized in ice-cold lysis buffer: 10 mM Tris HCl pH 7.4, 150 mM NaCl, 0.5 mM EDTA, 1% NP40, supplemented with EDTA-free proteinase inhibitor cocktail (cOmplete, Roche, 4693132001). Cell lysates were pre-cleared with 30 μl of Protein G Sepharose 4 Fast Flow (GE Healthcare, 17-0618-01). Total protein in the supernatants was used as input. IP was performed in 1000 μl lysis buffer containing 40 μl of slurry of ANTI-FLAG M2 Affinity Gel (Sigma, A2220). Samples were incubated overnight at 4°C, followed by three washes with 500 μl of TBS. Bead-tagged proteins were eluted using NuPAGE LDS sample buffer (Thermo Fisher, NP0007). After boiling at 95°C for 5 min, eluted proteins were separated on pre-cast gels (4-20% gradient; Genescript, M42015C). Immunoblotting was done using HA-Tag (C29F4) Rabbit mAb (Cell Signaling Technology,3724; 1:1000), monoclonal ANTI-FLAG M2 antibody (Sigma, F1804; 1:1000) and secondary goat anti-mouse antibodies conjugated to horseradish peroxidase (bioworld, BS12478 and BS13278, 1:5000). Chemiluminescence signals were detected by using the Tanon 5200 imaging system.

### Oil Red O staining

A 0.5% stock of Oil Red O (ORO) was made in 100% 2-propanol, shaken overnight, filtered and stored at 4°C. A working solution of 0.25% ORO in 60% 2-propanol was used for staining. Larvae were placed in microcentrifuge tubes and fixed in 4% paraformaldehyde (PFA) at 4°C overnight. The following day, larvae were rinsed twice with PBS and 0.1% Tween 20 (PBT). A solution of 60% 2-propanol was added and larvae were allowed to sit for 1 h. The solution was removed and the larvae were stained with ORO stain working solution for 75 min. They were then briefly rinsed with 60% 2-propanol and washed twice with 60% 2-propanol for 10 min. Lastly, larvae were rinsed twice with PBT and stored in 70% glycerol at 4°C.

### Histochemistry

Larvae and zebrafish liver were fixed in Dietrich's fixative (30% ethanol/2% glacial acetic acid/3.7% formaldehyde) for 24 h at room temperature, followed by three washes in PBT and paraffin embedding (Ellis and Yin, 2017). 4-μm-thick sections were acquired using the semi-automatic microtome HM 340 (Thermo Fisher) and stained with Hematoxylin and Eosin (H&E). For ORO staining, zebrafish liver and muscle tissues were fixed with 4% PFA at 4°C overnight and then transferred to a 30% sucrose solution until they sunk. Then samples were then embedded using OCT. Samples were cut into 10-µm-thick sections and ORO staining was performed as described above. For PAS staining (a method used to detect polysaccharides), zebrafish liver and skeleton muscles were fixed with 4% PFA at 4°C overnight, followed by two rinses with PBT. Then, samples were processed for paraffin embedding. After slides were deparaffined, they were rehydrated for PAS staining.

### Dissection of adult zebrafish thyroid gland

Based on a protocol previously described (Gillotay et al., 2020), the dissection of the adult zebrafish thyroid gland was performed by using ventral aorta as reference. Briefly, using fine forceps, the lower jaw was separated from the upper jaw and disconnected from the gut by pinching near the gills of euthanized zebrafish. The dissected tissue was carefully cleaned by removing muscle, skin, pectoral fin and lateral cartilages of the lower jaw, and fixed in 4% PFA overnight.

### Real-time qPCR

Total RNA was prepared using TRIzol (Invitrogen, 15596) and Direct-zol RNA Miniprep (Zymo Research, R2052) from control and *sec31a^nju221^* mutant samples. cDNA was synthesized with PrimeScript RT kit (Takara, RR047A). Real-time qPCR reactions were performed on the Roche LightCycler system using SYBR Green Master Mix (Takara, RR420A). Melt curves were examined to ensure primer specificity. Primers used in real-time qPCR were designed to span exon-exon junctions and are listed in Table S3.

### Measurements of blood and tissue chemistry parameters

Serum thyroxine (T4) levels were determined using a competitive ELISA kit (Invitrogen, EIAT4C) according to the manufacturer's instructions. The serum glycose levels were determined with the LABassay Glucose (Mutarotase-GOD method) kit (Wako, 638-50971). Triglyceride, total cholesterol and glycogen levels were measured using high-sensitivity triglyceride assay kits (Sigma-Aldrich, MAK264-1KT), total cholesterol assay kits (Nanjing Jiancheng Bioengineering Institute, A111-1) and glycogen assay kits (Abcam, ab65620), respectively, according to the manufacturer's instructions.

### Western blotting

Liver tissue was homogenized using RIPA lysis buffer (50 mM Tris-HCl, 1% NP-40, 0.25% sodium deoxycholate, 150 mM NaCl, 1 mM EDTA, 1 mM PMSF, 1 mM Na_3_VO_4_, and 1 mM NaF pH 7.4) containing 1% protease inhibitor cocktail (Roche, 11697498001) and then centrifuged for 15 min at 12,000 ***g*** at 4°C. The total protein concentration was measured using the Pierce BCA Protein Assay Kit (Thermo Fisher Scientific, 23225) according to the manufacturer's protocol. Equal amounts of total protein were loaded into each lane for SDS-PAGE, and then proteins were transferred to PVDF membranes. After blocking with 5% non-fat milk in TBS with 0.1% Tween 20, the membranes were incubated with primary antibodies against Dio1 (Santa Cruz Biotechnology, sc-515198, 1:100) and tubulin (Bioworld, BS1699, 1:1000), and subsequently with secondary antibodies conjugated with horseradish peroxidase (Bioworld, BS12478 and BS13278, 1:5000). Finally, the membranes were visualized with an ECL kit (Yeasen, 36208ES60) using the Tanon 5200 imaging analysis system (Tanon). ImageJ (v1.51; https://imagej.net/) was used for data quantification.

### Imaging

Whole imaging was performed using a Leica DFC320 camera on a Leica M205FA stereomicroscope. All confocal images were acquired using a Zeiss LSM880 confocal microscope. Image acquisition parameters (laser power and detector settings) were kept consistent within each experiment. The integrated density of GalT-TagBFP and mYFP was quantified using Fiji/ImageJ software.

### Survival curve

Eighty wild-type siblings and 80 *sec31a^nju221^* animals were put into 3 l tanks from 7 dpf. Every 3 days, the number of living fish was counted till 60 dpf. Kaplan–Meier curves were generated with GraphPad Prism 9 (Goel et al., 2010).

### Transcriptome sequencing

RNA libraries were constructed with NEBNext Single Cell/Low Input RNA Library Prep Kit for Illumina (New England Biolabs, 6420) and NEBNext Multiplex Oligos for Illumina (New England Biolabs, E7335). The libraries were sequenced on an Illumina Novaseq platform with the PE150 sequencing setting. Following quality control and pre-processing, HISAT2 v2.1.0 was used to map the sample sequencing reads to the GRCz11 reference genome (Kim et al., 2019). Gene expression counts were calculated using FeatureCounts v1.6.0 (Liao et al., 2014) based on the current Ensembl annotation. All downstream data analyses were carried out in R (v4.3.2) or RStudio. The DESeq2 package (v1.42.1) was used for differential analysis of count data, where low total normalized read counts across all samples (<10) were filtered out from the dataset (Love et al., 2014).

### Quantitative proteomic analysis

Liver tissues dissected from adult *sec31a^nju221^* and wildtype zebrafish were lysed in RIPA lysis buffer [50 mM Tris-HCl, 1% NP-40, 0.25% Na-deoxycholate, 150 mM NaCl, 1 mM EDTA, protease inhibitor cocktail tablet (Roche, 04693132001), 1 mM PMSF, 1 mM Na_3_VO_4_, and 1 mM NaF, pH 7.4], ultrasonicated by Bioruptor Plus (Diagenode, Liege) for 10 min at 0°C, and centrifuged at 13,000 ***g*** at 4°C for 15 min to remove debris. The protein concentration in each sample was determined using the Pierce BCA Protein Assay Kit and the total protein concentrations were adjusted to be 1 μg/μl. For on-filter digestion, an aliquot of total protein (50 μg) was reduced with 5 mM tris(2-carboxyethyl)phosphine (TCEP) for 1 h at 55°C and alkylated with 6.25 mM methyl methanethiosulfonate (MMTS) for 30 min at room temperature in darkness. The protein lysates were transferred to 3 kDa Vivacon filters following centrifugation at less than 9600 ***g*** for 30 min to remove the solvent, and washed with 8 M urea three times and 1 M tetraethylammonium bromide (TEAB) six times. Samples were then supplemented with trypsin (Promega, V5280) for 4 h pre-digestion (enzyme-to-substrate mass ratio 1:50) at 37°C, followed by an additional 8 h digestion with trypsin/LysC (Promega, V5073, enzyme-to-substrate mass ratio 1:100) at 37°C. After C18 desalting and vacuum drying, the digested peptides were resuspended in 3% acetonitrile (v/v) and 2% formic acid (v/v) for liquid chromatography-tandem mass spectrometry analysis. The samples were acquired in DIA mode with the 55-min microflow gradient on a ZenoTOF 7600 mass spectrometer (Sciex, MO, USA). The DIA data analysis was conducted using DIA-NN (Demichev et al., 2020) for quantitative proteomic analysis and searched against the *Danio rerio* UniProt database (accessed 2 February 2023, containing 46,122 sequences, http://www.uniprot.org/proteomes/UP000000437). The mass spectrometry proteomics data have been deposited to the ProteomeXchange Consortium (Deutsch et al., 2023) via the PRIDE (Perez-Riverol et al., 2022) partner repository with the dataset identifier PXD048925.

### Pharmacological treatments

#### Embryos

Dechorionated embryos were randomly distributed in 12-well plates (20 embryos/well, 2 ml E3 medium/well). Embryos were treated with 10 nM L-thyroxine (MedChemExpress, HY-18341) from gastrula stage (∼5.3 hpf) to 7 dpf, or with 5 μM GSK2606414 (MedChemExpress, HY-18072) or 10 μM GCN2-IN-1 (MedChemExpress, HY-100877) from 24 hpf to 7 dpf. The solvent, DMSO, was used as the vehicle control. The drugs were added to the E3 medium at these concentrations and were replaced daily.

#### Adult fish

A 4-week treatment with L-thyroxine was sustained in a closed system that closely resembled aquarium conditions. Three groups including untreated wild-type siblings, vehicle-treated *sec31a^nju221^* fish and *sec31a^nju221^* fish treated with L-thyroxine were subject to this regime, with each group comprising twelve adult fish. L-thyroxine was added three times a week at a concentration of 30 nM. Water was changed three times each week.

### Bioinformatics analysis

We used the following software and tool: Gene Set Enrichment Analysis (GSEA) (https://www.gsea-msigdb.org/gsea/index.jsp), a computational method for determining whether an a priori defined genome is statistically significant. Molecular Signature Database (MSigDB) (https://www.gsea-msigdb.org/gsea/msigdb/), a resource of gene sets and specific biological processes that are significantly differentially expressed in different groups. Analyses using MSigDB resulted in statistically significant improvements in associations between data expression patterns and biological processes, ignoring thresholds for significantly different genes. 1000 alignments were performed for each genome. Normalized enrichment score (NES) and false discovery rate (FDR) values were used to explore enrichment pathways for each phenotype. *P*<0.05 and FDR<0.25 were used as thresholds.

Metascape (https://metascape.org/gp/index.html#/main/step1) (Yingyao Zhou et al., 2019), an analytical website that integrates functional enrichment, genetic annotation and transcriptional regulatory networks, utilizing more than 40 individual knowledge bases in a comprehensive portal. Gene Ontology (GO) Resource (https://geneontology.org/) is a major bioinformatics initiative for high-quality functional gene annotation. Transcription factor–target regulatory interactions can also be inferred from high-throughput gene expression data using a wide variety of computational algorithms. The gene annotation and analysis resource Metascape (https://metascape.org/gp/index.html#/main/step1) was used to predict the functions of genes listed in the Database of Essential Genes (DEG) (http://origin.tubic.org/deg/public/index.php), with the screening conditions set as minimal overlap =3 and minimal enrichment =1.5, with *P*<0.05 to be considered statistically significant.

### Statistical analysis

Statistical analyses were performed using GraphPad Prism 9. One-way ANOVA analysis was followed by Tukey's multiple comparisons test for measurements of blood and tissue chemistry parameters with L-thyroxine treatments. Other statistical tests were performed using unpaired two-tailed Student's *t*-tests. Numerical data are presented as mean±s.e.m. Differences were considered significant if *P*<0.05 and highly significant if *P*<0.01. All experiments were carried out with at least three biological replicates. The numbers of animals used are described in the corresponding figure legends.

## Supplementary Material

10.1242/dmm.050748_sup1 Supplementary information

Table S4. Proteomics dataset for adult zebrafish liver.
