# Supplementary material for: Compromised COPII vesicle trafficking leads to glycogenic hepatopathy
Source: Dis Model Mech. 2024 Sep 30;17(9):dmm050748. doi: 10.1242/dmm.050748 (PMC11463966; doi:10.1242/dmm.050748)
Supplement: Supplementary information [file dmm-17-050748-s1.pdf]

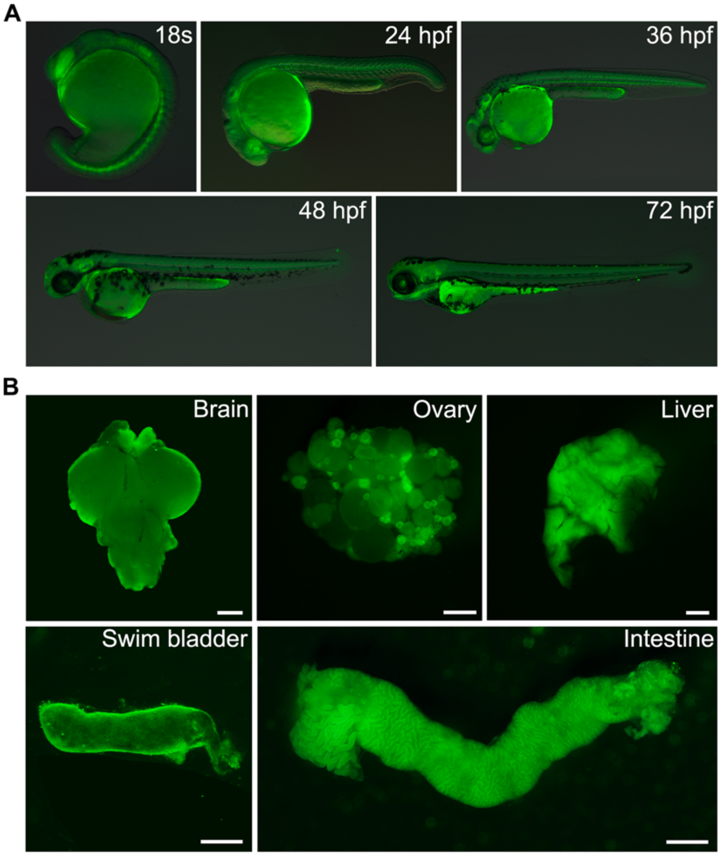

Fig. S1. GFP expression pattern of gene trap line NT-1254 in embryo and adult fish.

(**A**) At 18-somite stage, GFP signal emerged in primordia of eyes, otic placode and notochord. At 36 hpf, GFP signal is evident in regions of brain, lens, otic vesicles and notochord. As development proceeded, strong GFP expression could be observed in pharyngeal arches and liver.

(**B**) At adult stage, GFP signal could be observed in brain, ovary, liver, swim bladder and intestine. Scale bars: 600 μm.

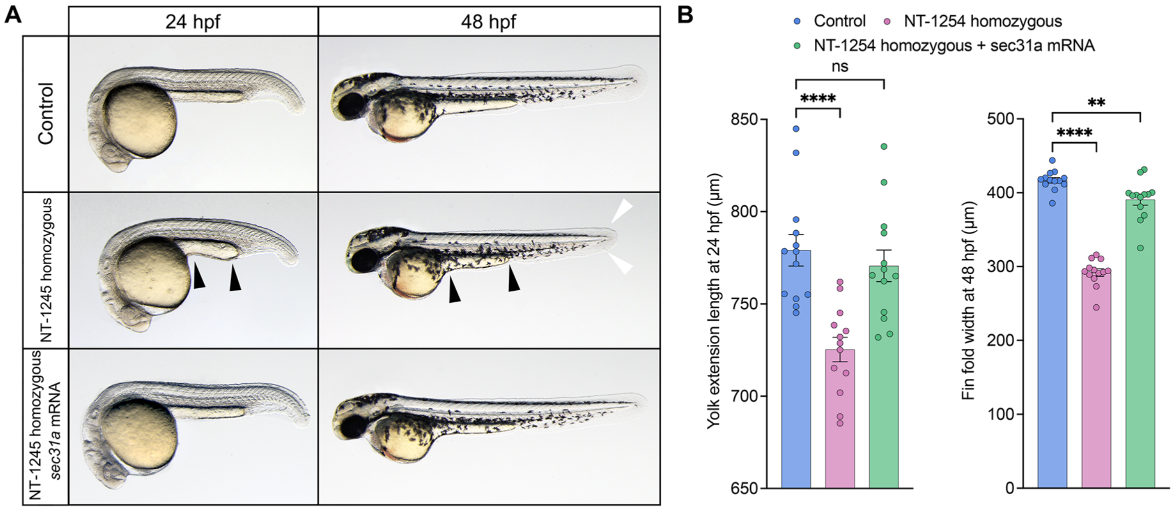

Fig. S2. Injection of *sec31a* mRNA rescues the early developmental defects of NT-1254 homozygous embryos.

(**A**) Upper panel, live images of wild type zebrafish embryos. Middle panel, live images of homozygous mutant embryos from incrosses of heterozygous NT-1254 fish. Black arrowheads indicate the disformed yolk extension. White arrowheads indicate impaired fin fold growth. Lower panel, live images of NT-1254 homozygous embryos injected with *sec31a* mRNA.

(**B**) The statistical data for yolk extension and fin fold growth phenotype displayed in **A**. n = 13 for each genotype group.

Disease Models & Mechanisms • Supplementary information

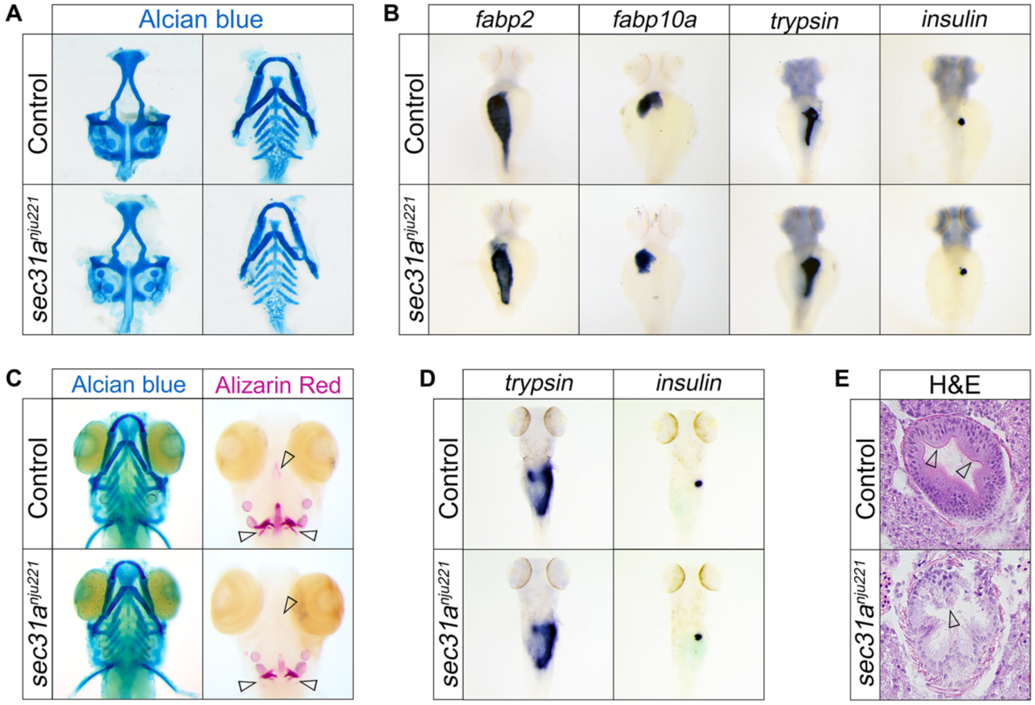

Fig. S3. Development of head skeleton and digestive organs are largely normal in *sec31a*^{nju221} embryos.

(**A**) Alcian blue staining showing the cartilage elements in head skeleton. Ventral views at 4 dpf.

(**B**) RNA *in situ* hybridization for marker genes specifically for the intestine (fabp2), liver (fabp10a) , exocrine pancreas (trypsin), and endocrine pancreas (insulin) on 3 dpf embryos.

(**C**) Alcian blue staining of head cartilage and Alizarin red staining of cranial bones. Ventral views at 7 dpf. Arrowheads indicate the missing bone fragments in *sec31a*^{nju221} embryos.

(**D**) RNA *in situ* hybridization for marker genes specifically for the exocrine pancreas (trypsin) and endocrine pancreas (insulin) on 7 dpf embryos.

(**E**) Hematoxylin and eosin (H&E) staining of intestine from 7 dpf zebrafish larvae. Arrowheads indicate the defective intestine epithelium in *sec31a*^{nju221} embryos.

Disease Models & Mechanisms • Supplementary information

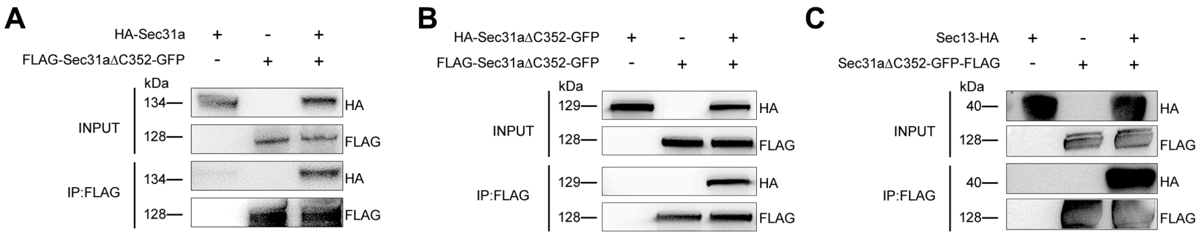

Fig. S4. Co-immunoprecipitation analysis for Sec31aΔC352-GFP protein.

(**A**) HEK-293T cells were transfected with HA-Sec31a and FLAG-Sec31aΔC352-GFP plasmids.

(**B**) HEK-293T cells were transfected with HA-Sec31aΔC352-GFP and FLAG-Sec31aΔC352-GFP plasmids.

(**C**) HEK-293T cells were transfected with Sec13-HA and Sec31aΔC352-GFP-FLAG plasmids. For **A-C**, An anti-FLAG antibody was used to immune-precipitate the tagged proteins. The input and the IP products were blotted using an anti-HA antibody or anti-FLAG antibody respectively.

Disease Models & Mechanisms • Supplementary information

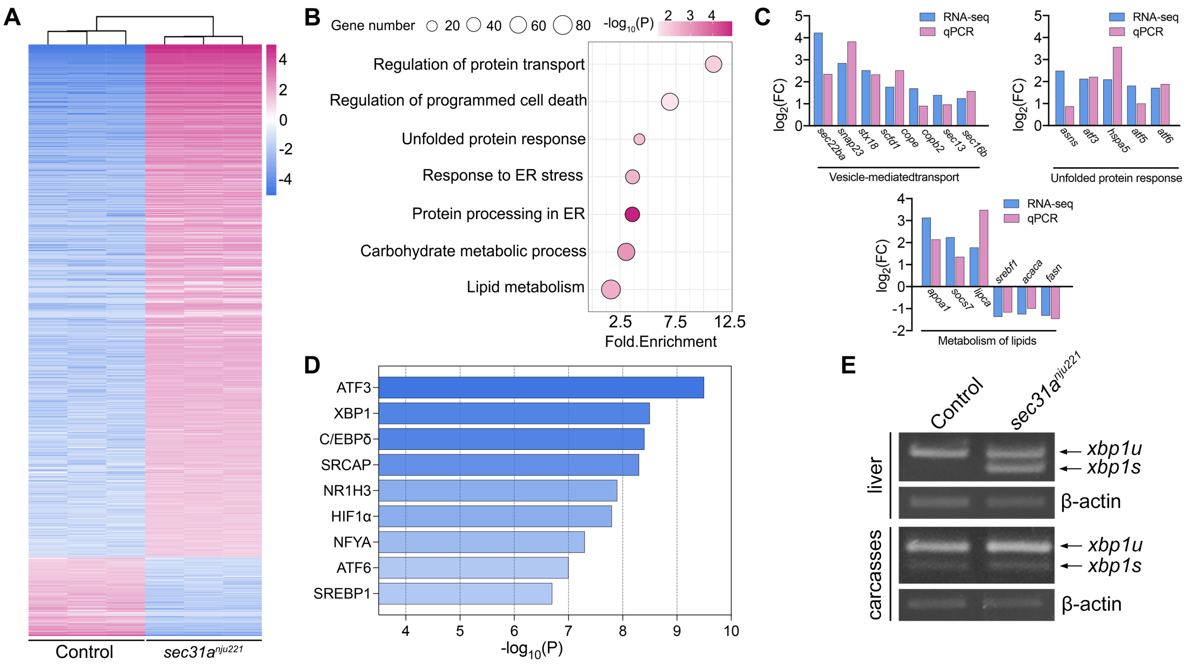

Fig. S5. Transcriptome profiling revealed activation of UPR in *sec31a^{nju221}* livers.

(**A**) Heat map representation of genes differentially expressed in livers of *sec31a^{nju221}* larvae at 5 dpf. Magenta, upregulated; blue, downregulated; white, no significant change.

(**B**) Bubble plot showing the enrichment for gene ontology (GO) of differentially expressed genes (DEG) in livers of *sec31a^{nju221}* larvae. The size of the dots represents the number of different genes in the corresponding biological process and molecular function term.

(**C**) Comparison of the changes in expression of selected genes measured by RNA-seq and qPCR.

(**D**) Summary of putative regulators of DEG were determined with Metascape and the TRRUST database.

(**E**) PCR analysis of *xbp1* splicing with primers for the detection of both unspliced (*xbp1u*) and spliced forms (*xbp1s*). Data revealed robust splicing in 5-dpf *sec31a^{nju22}* livers and comparable splicing in the liverless carcass. The data are representative of three experiments.

Disease Models & Mechanisms • Supplementary information

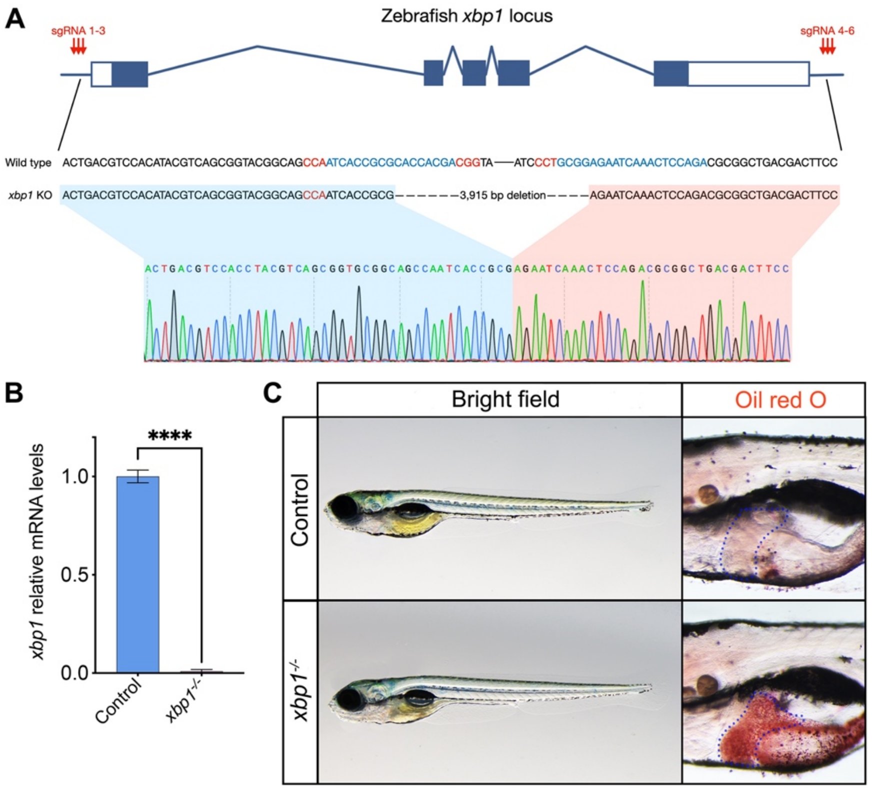

Fig. S6. Generation of *xbp1* knockout zebrafish line.

(**A**) The zebrafish *xbp1* genomic locus and Cas9/sgRNA targeting site. Deletions in Δ3915 allele are shown as dashes.

(**B**) QPCR results showing the absence of *xbp1* mRNA in knock out embryos. Data are mean ± SEM. ****P < 0.0001.

(**C**) Left panels, live images of control and *xbp1*$^{-/-}$ zebrafish at 5 dpf. Right panels, Oil Red O (ORO) staining of 7 dpf zebrafish larvae, livers were outlined with dashed line. Lateral view, anterior to the left.

Disease Models & Mechanisms • Supplementary information

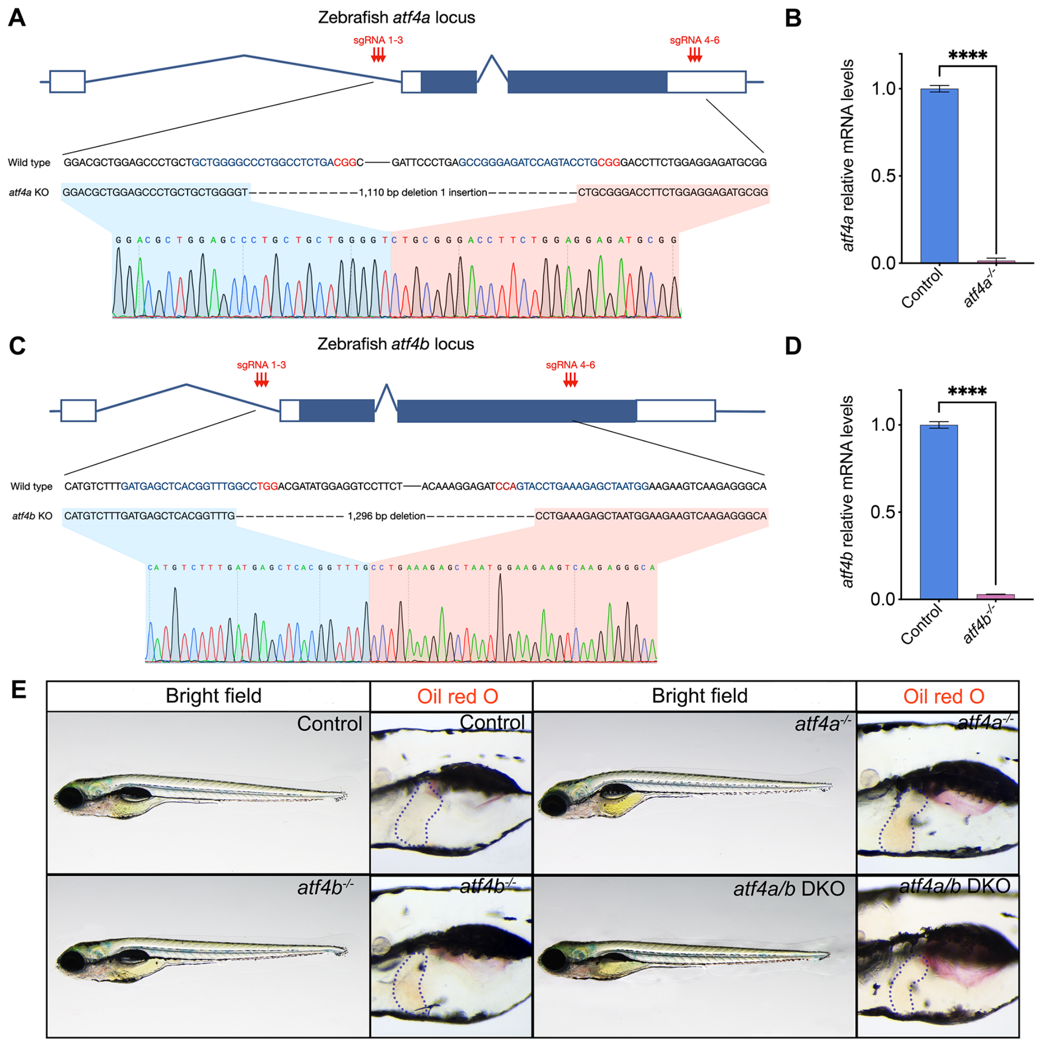

Fig. S7. Generation of *atf4a* and *atf4b* knockout zebrafish line.

(**A**) The zebrafish *atf4a* genomic locus and Cas9/sgRNA targeting site. Deletions in Δ1110 allele are shown as dashes.

(**B**) QPCR results showing the absence of *atf4a* mRNA in knock out embryos. Data are mean ± SEM. ****P < 0.0001.

(**C**) The zebrafish *atf4b* genomic locus and Cas9/sgRNA targeting site. Deletions in Δ1296 allele are shown as dashes.

(**D**) QPCR results showing the absence of *atf4b* mRNA in knock out embryos. Data are mean ± SEM. ****P < 0.0001.

(**E**) Live images and ORO staining of control and knock out zebrafish at 7 dpf. For ORO staining, livers were outlined with dashed line. Lateral view, anterior to the left.

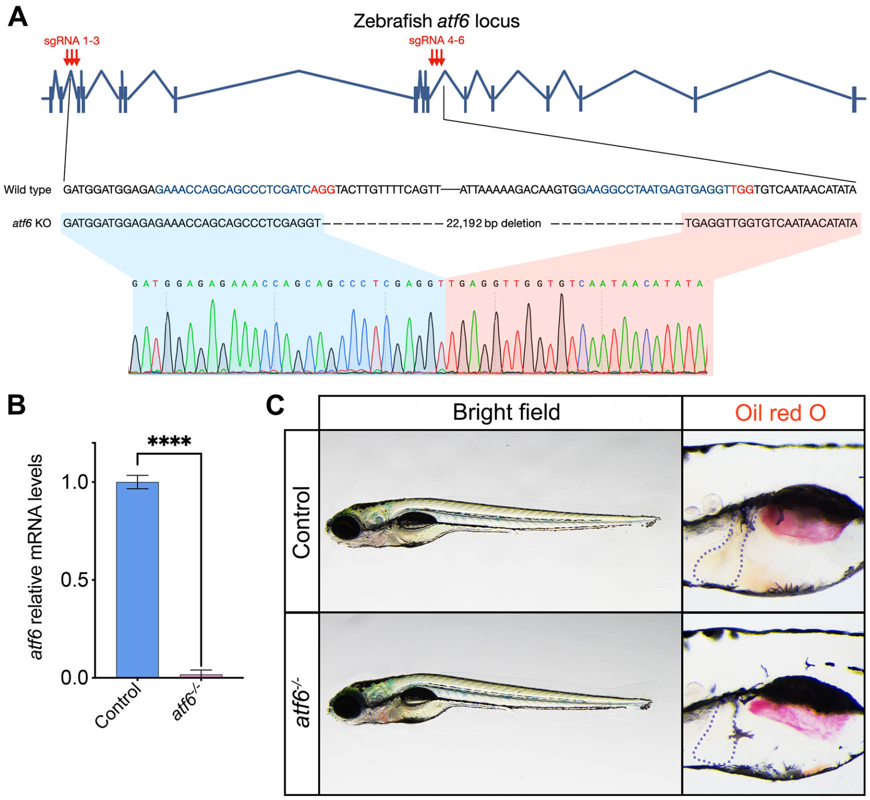

Fig. S8. Generation of *atf6* knockout zebrafish line.

(**A**) The zebrafish *atf6* genomic locus and Cas9/sgRNA targeting site. Deletions in Δ22192 allele are shown as dashes.

(**B**) QPCR results showing the absence of *atf6* mRNA in knock out embryos. Data are mean ± SEM. ****P < 0.0001.

(**C**) Left panels, live images of control and *atf6*$^{-/-}$ zebrafish at 5 dpf. Right panels, Oil Red O (ORO) staining of 7 dpf zebrafish larvae, livers were outlined with dashed line. Lateral view, anterior to the left.

Disease Models & Mechanisms • Supplementary information

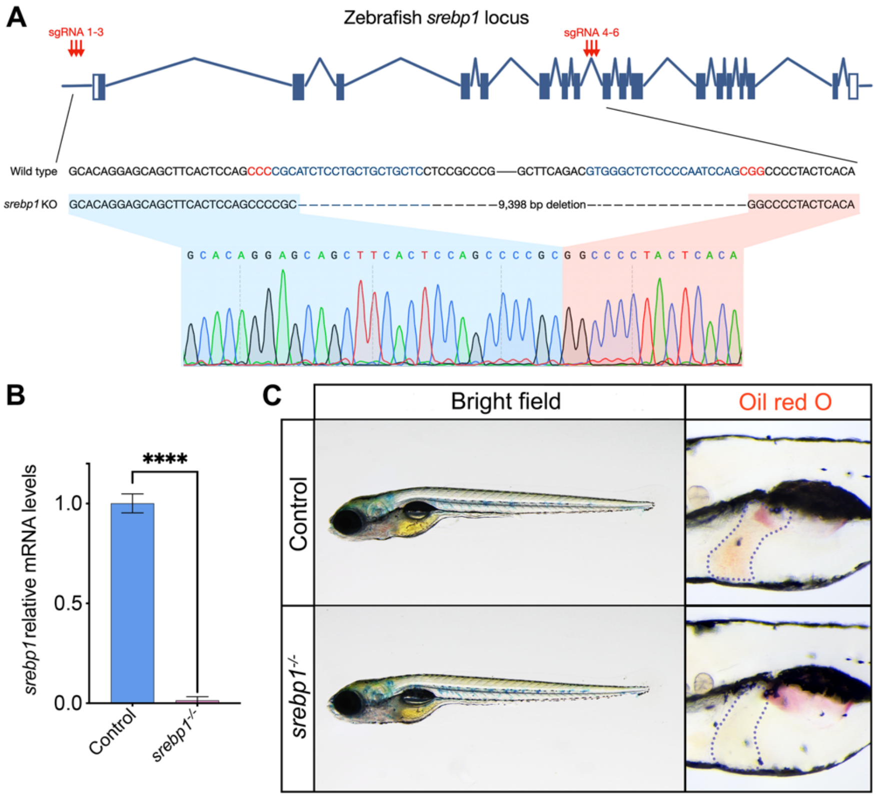

Fig. S9. Generation of *srebp1* knockout zebrafish line.

(**A**) The zebrafish *srebp1* genomic locus and Cas9/sgRNA targeting site. Deletions in Δ9398 allele are shown as dashes.

(**B**) QPCR results showing the absence of *srebp1* mRNA in knock out embryos. Data are mean ± SEM. ****P < 0.0001.

(**C**) Left panels, live images of control and *srebp1*$^{-/-}$ zebrafish at 5 dpf. Right panels, Oil Red O (ORO) staining of 7 dpf zebrafish larvae, livers were outlined with dashed line. Lateral view, anterior to the left.

Disease Models & Mechanisms • Supplementary information

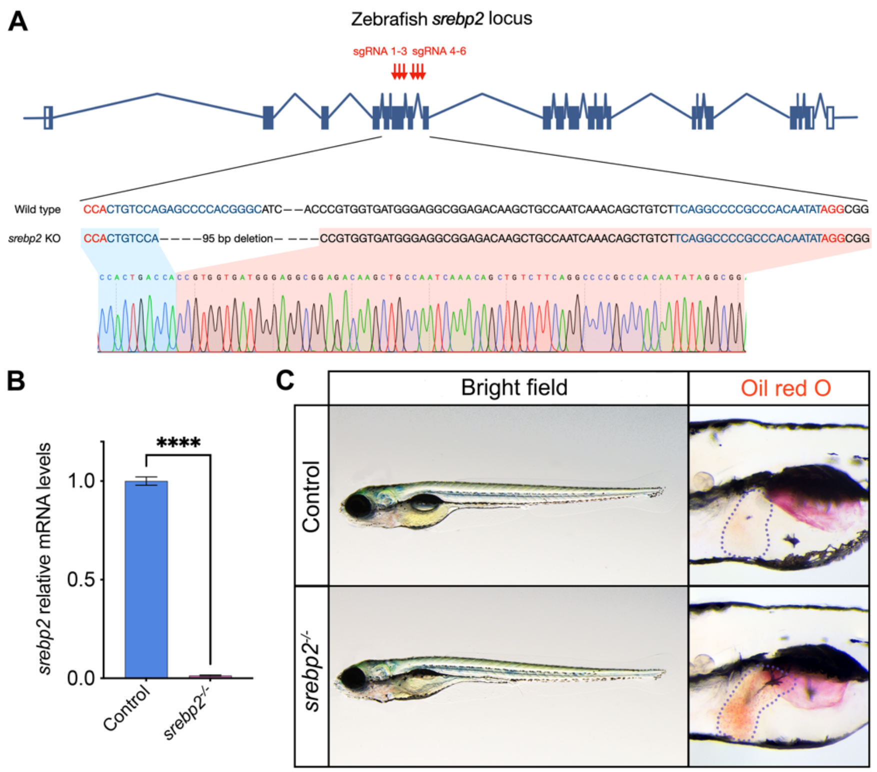

Fig. S10. Generation of *srebp2* knockout zebrafish line.

(**A**) The zebrafish *srebp2* genomic locus and Cas9/sgRNA targeting site. Deletions in Δ95 allele are shown as dashes.

(**B**) QPCR results showing the absence of *srebp1* mRNA in knock out embryos. Data are mean ± SEM. ****P < 0.0001.

(**C**) Left panels, live images of control and *srebp2*$^{-/-}$ zebrafish at 5 dpf. Right panels, Oil Red O (ORO) staining of 7 dpf zebrafish larvae, livers were outlined with dashed line. Lateral view, anterior to the left.

Disease Models & Mechanisms • Supplementary information

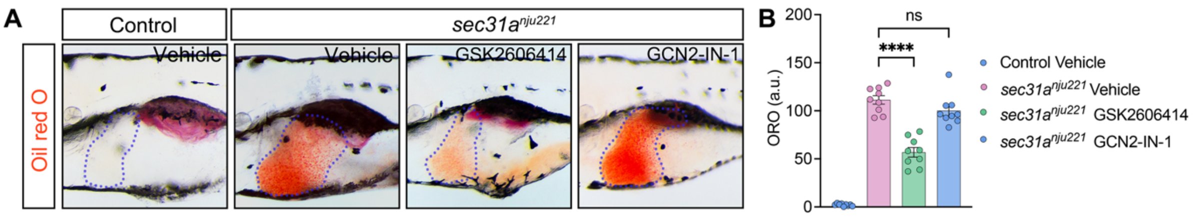

Fig. S11. Inhibition of PERK alleviates hepatic steatosis of sec31a^{nju221} larvae.

(**A**) Oil Red O staining for 7 dpf zebrafish larvae which were treated with vehicle, 5uM GSK2606414 or 10 μM GCN2-IN-1 from 24 hpf. Livers were outlined with dashed line.

(**B**) Quantification of Oil red O staining intensity in **A**. Data are the mean ± SEM, **** indicates p<0.0001, Student's t-test.

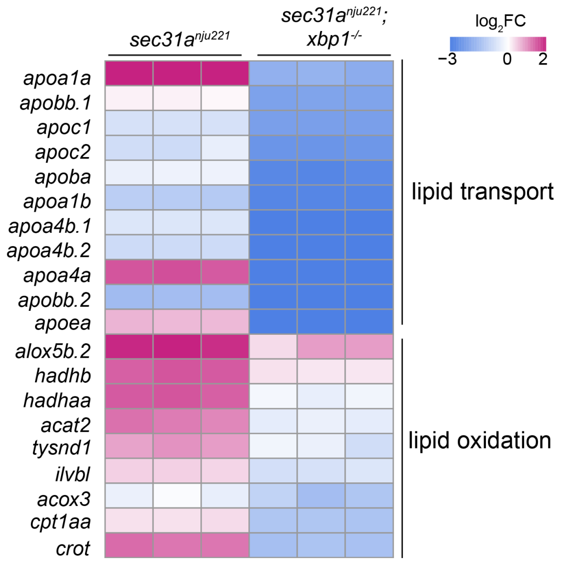

Fig. S12. Heatmap showing the expression levels of genes involved in lipid and transport oxidation in liver from *sec31a^{nju221}* and *sec31a^{nju221}*; *xbp1$^{-/-}$* larvae.

Disease Models & Mechanisms • Supplementary information

Table S1. Quantification of liver oil red O staining on zebrafish knock-out lines

Genotype	Oil red O Integrated density (a.u.)	Genotype	Oil red O integrated density (a.u.)
Wild type	2.5 ± 1.4		
sec31a^{nju221}	73.3 ± 1.0		
sec31a^{nju221}; *atf4a/b* DKO	33.0 ± 2.3 (****)[a]	*atf4a/b* DKO	5.6 ± 0.6 (ns)[b]
sec31a^{nju221}; *xbp1*$^{-/-}$	77.6 ± 0.4 (ns)[a]	*xbp1*$^{-/-}$	50.3 ± 2.7 (****)[b]
sec31a^{nju221}; *atf6*$^{-/-}$	65.4 ± 1.3 (**)[a]	*atf6*$^{-/-}$	5.5 ± 0.1 (ns)[b]
sec31a^{nju221}; *srebp1*$^{-/-}$	75.4 ± 0.4 (ns)[a]	*srebp1*$^{-/-}$	23.5 ± 1.3 (****)[b]
sec31a^{nju221}; *srebp2*$^{-/-}$	71.6 ± 0.8 (ns)[a]	*srebp2*$^{-/-}$	54.2 ± 2.1 (****)[b]

Staining signal were determined and analyzed using ImageJ software. a.u., arbitrary units. Data represent the mean ± s.e.m. * p ≤ 0.05; ** p ≤ 0.01 ****; p ≤ 0.0001; ns = no significant. [a], compared with *sec31a*^{nju221} samples. [b], compared with wild-type samples.

Disease Models & Mechanisms • Supplementary information

Table S2. Primers for genotyping crispr/cas9 knockout fish lines

Genes	Forward Primer (5′-3′)	Reverse Primer (5′-3′)	Amplicon size from WT allele	Amplicon size from KO allele
atf4a	CTCCAGTGATTAAACCTGATGAAGG	GAGATGCAGGATGAGGAGTTATGATG	1447 bp	339 bp
	GTCACCCCTTTGCTAAATGACTG	GTGTATTGGTCACCTGTGCTCTG	405 bp	-
atf4b	CAGGTCCTTTGTGGCAACTCTGATC	CAGTGCATAGGATAAGATACACACGAC	1711 bp	415 bp
	GGGCTGATGGTGTGATTATGAG	CATCTTTATCTAGAATTGACCGCAG	327 bp	-
atf6	GTTGTCATCCATTGCTAATGTATTGCTCAC	GAAAAAAGCAGGACTTCAGTTCCATCC	22790 bp	595 bp
	CACACCGAACTGAACTTCAACTC	GGACACAGCAACATAATACCCC	369 bp	-
srebf1	GATTTGATTAGGCAGGAATAACTGGACTG	GTGTGTGTGTGTGTGTTACCTTGTTG	10070 bp	687 bp
	GATTTGATTAGGCAGGAATAACTGGACTG	GCGGCTGGAAGGCTTTATCT	733 bp	-
srebf2	ATGCAGAGTATCATGACGTCTCCG	GTTCACCTGCTGCAGGTATTTGATG	1072 pb	977 bp
xbp1	CTCAGCCTCCTTTGTTTATGTTGTG	GTCAGATACGAAGCCTTCTCGTAGC	4309 bp	394 bp
	CTAAGTGCCCTGTGAAATTCCTATC	CTTCTGTTTTGATTAGGGGTGGG	477 bp	-

Disease Models & Mechanisms • Supplementary information

Table S3. Primers for quantitative real-time RT-PCR analysis

Genes	Forward Primer (5′-3′)	Reverse Primer (5′-3′)
pcxb	CCTTCAGGTCAAAGGTGCTC	GTGATTTCATCCCCGTGTGC
hpxa	GGTGCCCTCACTTTGGGAG	GCCAGCGTCGTCAGATGTTA
ces2a	ATGCCACCGAACACCCTTTA	CAGACCCCCTCCATGAATCC
ces2b	AGCGCTCCTTTCACAGAAGA	TTTTCCCAGGTTTCTGTTGC
sec22ba	TCACTTTCTGCCAGTCACTCAA	GTCTCGTCCAGACTGCTCGT
snap23	CGGCCCCTATATCACTAGAATAA	ATTTCCAGTGTTTGGTCCCT
stx18	GAACGCCGGCAGTGTGATGAGC	CTTTTTGTCCATCTCGGAGCG
scfd1	CTGCTCTCAAACGAATGCTC	TCTGAGTGCAACAAGAGGTG
cope	GGCAAACCATTTGAGGTTACA	GTATTATTTCAGGCGCTGGG
copb2	GGTCATAACTGGCGCTGATG	ACCTGACTGCACGACCATTT
sec13	CCCATGTACGGCAACATCCT	GCAAACCGAATTGACCGAGG
sec16b	GCTTTGACAACCAAAGAATCCTC	CACTCCACATTATCAGTTGGAACA
asns	GGGAAAGTGGAGTCTGTTCA	TGTCTCCAATTCGAAGTCTGT
atf3	CACACAACCAGATTTCGGCAT	CGTTGGACATGCGTTTGTTCT
hspa5	TCATCAATGAGCCTACGGCG	GTGGCCACCACTTCAAACAC
atf5a	GAAGACTCTACTCGTCTGCC	GTCACTAAGACCATCACCAATTA
atf6	CTGTGGTGAAACCTCCACCT	CATGGTGACCACAGGAGATG
apoa1	TCTCTTGGCCTTGGGTTCC	GCTCATAGTCGGTTCCGTCC
socs7	TCAGTCTGCTGGATGCTTTC	AGTGAAGCTACTGGAATCGG
lipca	ATTCATGGCTGGTCGGTAGA	GAGTCAGCCAATCTGCAATC
srebf1	CATCCTGACCACCGTTCCTC	TCGGTAGCGTTTCTCGATGG
acaca	TGCTGAGAGTGAGGACAGAATG	TGACAGGGTGGGGTAAAGGA
fasn	CTACAAACTGCACGAGGTGTG	TTTATGCGAAGGTTTAGCCCTCT
xbp1	GAGGAGCCCACAAAGTCCTC	CGAAGTGCTTTTTCCTCTGG
srebp1	CATCCTGACCACCGTTCCTC	TCGGTAGCGTTTCTCGATGG
srebp2	CTCTAAGCCCCTCCCAGACT	GGGGTCCGCTTTATCTCTCG
atf4a	TTAGCGATTGCTCCGATAGC	GCTGCGGTTTTATTCTGCTC
atf4b	CTGCCCTGGTTACCCTCAGA	TTCCACTAAACGAATGATCTTCACC
18s	TCGCTAGTTGGCATCGTTTATG	CGGAGGTTCGAAGACGATCA

Disease Models & Mechanisms • Supplementary information

Table S4. Proteomics dataset for adult zebrafish liver.

Available for download at

https://journals.biologists.com/dmm/article-lookup/doi/10.1242/dmm.050748#supplementary-data

Table S5. Zebrafish lines used in this study.

Fish line	Source	Genotype
AB	China Zebrafish Resource Center	Wild type
sec31a^{nju221}	This paper	*sec31a*^{nju221}
xbp1$^{-/-}$;*sec31a*^{nju221}	This paper	*xbp1*$^{-/-}$;*sec31a*^{nju221}
atf4a$^{-/-}$;*sec31a*^{nju221}	This paper	*atf4a*$^{-/-}$;*sec31a*^{nju221}
atf4b$^{-/-}$;*sec31a*^{nju221}	This paper	*atf4b*$^{-/-}$;*sec31a*^{nju221}
atf4a/b dko;sec31a^{nju221}	This paper	*atf4a*$^{-/-}$;*atf4b*$^{-/-}$;*sec31a*^{nju221}
atf6$^{-/-}$;*sec31a*^{nju221}	This paper	*atf6*$^{-/-}$;*sec31a*^{nju221}
srebp1$^{-/-}$;*sec31a*^{nju221}	This paper	*srebp1*$^{-/-}$;*sec31a*^{nju221}
srebp2$^{-/-}$;*sec31a*^{nju221}	This paper	*srebp2*$^{-/-}$;*sec31a*^{nju221}

Disease Models & Mechanisms • Supplementary information
